# Reviewed article: Carvalho EA, Hopker RH, Pedroso GH, Almeida LS, Pacher JAT, Antônio ALM, Souza J, Zeny MS, Santos MLSF, Valle DAV, Andrade FA. Characterization of patients treated at a rare disease referral service: a descriptive study, 2016-2021. Epidemiol Serv Saúde. 2024;33:e20240204. 10.1590/S2237-96222024v33e20240204.en

**DOI:** 10.1590/S2237-96222024v33e20240204.b

**Published:** 2024-12-20

**Authors:** Cassandra de Sousa Costa

**Affiliations:** Instituto de Medicina Integral Professor Fernando Figueira, Recife, PE, Brasil

## Peer review B

## Questionnaire

Does the manuscript contain new and significant information to justify publication? Yes

Does the Abstract (Summary) clearly and accurately describe the content of the article? Yes

Is the problem significant and concisely stated? Yes

Are the methods described comprehensively? Yes

Are the interpretations and conclusions justified by the results? Yes

Is adequate reference made to other work in the field? Yes

## Manuscript Structure

Length of article is: Adequate

Number of tables is: Adequate

Number of figures is: Adequate

**Please state any conflict(s) of interest that you have in relation to the review of this paper (state “none” if this is not applicable).**

None

**Interest:** Good

**Quality:** Good

**Originality:** Excellent

**Overall:** Good

**Recommendation:** Accept

## Comments

Tanto no resumo quanto em sua discussão, o autor relata que a eficácia da implementação da política pública de doenças raras pode estar associada a um menor tempo médio de diagnóstico, observada em seu estudo, em relação ao preconizado em sociedade internacional. A avaliação de eficiência da implementação de um programa de saúde envolve uma pesquisa de avaliação em saúde, com metodologia própria, que não pode ser aferida na pesquisa em questão. Pode-se observar que os resultados obtidos no presente estudo indicam os efeitos positivos advindos da implementação do serviço de referência, mesmo que com pouco tempo de atuação e complexidade inerentes às doenças raras.

Ainda em sua discussão, o autor cita que “curiosamente”, os pacientes que residiam no interior do Paraná enfrentam um maior atraso diagnóstico. Neste caso, há uma relação direta de causa e efeito, não somente relacionado às doenças raras mas em relação ao acesso aos serviços de saúde pelo SUS, em âmbito nacional. Porém no que tange essas doenças, pode apresentar maior dificuldade de acesso devido a especificidade das doenças raras. Este fato apresentou um resultado relevante na pesquisa do autor e pode ser melhor discutido em seu artigo.

